# Software for Dosage Individualization of Voriconazole: a Prospective Clinical Study

**DOI:** 10.1128/AAC.02353-18

**Published:** 2019-03-27

**Authors:** William Hope, Gary Johnstone, Silvia Cicconi, Timothy Felton, Joanne Goodwin, Sarah Whalley, Anahi Santoyo-Castelazo, Virginia Ramos-Martin, Jodi Lestner, Leah Credidio, Aaron Dane, Daniel F. Carr, Munir Pirmohamed, Rahim Salim, Michael Neely

**Affiliations:** a Antimicrobial Pharmacodynamics and Therapeutics, University of Liverpool, Liverpool, United Kingdom; b Royal Liverpool Broadgreen University Hospital Trust, Liverpool, United Kingdom; c Liverpool Clinical Trials Unit, University of Liverpool, Liverpool, United Kingdom; d Division of Infection, Immunity and Respiratory Medicine, School of Biological Sciences, Faculty of Biology, Medicine, and Health, The University of Manchester, Manchester Academic Health Science Centre, Wythenshawe, Manchester, United Kingdom; e Manchester University NHS Foundation Trust, Manchester Academic Health Science Centre, Wythenshawe Hospital, Wythenshawe, Manchester, United Kingdom; f DaneStat Consulting, Macclesfield, United Kingdom; g MRC Centre for Drug Safety Science, University of Liverpool, Liverpool, United Kingdom; h Laboratory of Applied Pharmacokinetics, University of Southern California, Los Angeles, California, USA

**Keywords:** antifungal agents, antifungal therapy, mathematical modeling, pharmacokinetics, population pharmacokinetics, software, voriconazole

## Abstract

Voriconazole is a first-line antifungal agent. Therapeutic drug monitoring is a standard of care. The best way to adjust dosages to achieve desired drug exposure endpoints is unclear due to nonlinear and variable pharmacokinetics. Previously described software was used to prospectively adjust voriconazole dosages. The *CYP2C19*, *CYP3A4*, and *CYP3A5* genotypes were determined. The primary endpoint was the proportion of patients with a *C*_min_ at 120 h in the range 1 to 3 mg/liter using software to adjust voriconazole dosages. A total of 19 patients were enrolled, and 14 were evaluable. Of these, 12/14 (85.7%; 95% confidence interval = 57.2 to 98.2%) had a *C*_min_ at 120 h posttreatment initiation of 1 to 3 mg/liter, which was higher than the *a priori* expected proportion of 33%. There was no association of *CYP* genotype-derived metabolizer phenotype with voriconazole AUC. Software can be used to adjust the dosages of voriconazole to achieve drug exposures that are safe and effective. (The clinical trial discussed in this paper has been registered in the European Clinical Trials Database under EudraCT no. 2013-0025878-34 and in the ISRCTN registry under no. ISRCTN83902726.)

## Abstract (precis)

Voriconazole is a first-line antifungal agent. Therapeutic drug monitoring is a standard of care.

## INTRODUCTION

Voriconazole is a first-line agent for the treatment of invasive mycoses that includes diseases caused by *Aspergillus* spp. (1), *Fusarium* spp. (2), and Scedosporium apiospermum complex (2). Although the efficacy of voriconazole has been repeatedly demonstrated in a wide range of clinical contexts, the utility of this agent is compromised by considerable pharmacokinetic variability, which is caused by nonlinear pharmacokinetics, variable oral bioavailability, and well-defined genotypic determinants (3, 4). This, coupled with detailed knowledge of drug exposure response and toxicity relationships (5, 6), has contributed to recommendations for therapeutic drug monitoring as a standard of care (7). However, there are no recommendations when to measure concentrations and how voriconazole dosages should be altered to achieve the desired concentration targets for individual patients.

The potential advantages for dosage individualization include improved efficacy, reduced toxicity, and suppression of the emergence of resistance. An additional benefit may include the use of less drug over the course of therapy. Currently, crude dosage adjustments are advocated, such as increasing the dose by 50%, which are potentially imprecise, leaving 30 to 50% of adult patients who fail to achieve a target concentration, depending on the target range (8). Some of the potential software tools that are available for dosage individualization are summarized elsewhere (9). Although there are methodologic and statistical differences between software tools, they all use a pharmacokinetic (PK) model that is based upon preexisting PK data to summarize prior knowledge of PK behavior. This model is then combined with a new, individual patient’s plasma PK data to design subsequent dosing that is most likely to achieve a desired drug exposure target in that patient. The different software tools have strengths and limitations regarding ease of use, capability, prediction accuracy and precision, and validation through published peer-reviewed investigations.

We have previously described the construction and validation of software that can be used to individualise the dosing of voriconazole in both children and adults (10, 11). The models were developed using nonparametric statistical population methods from prospectively conducted PK studies in children and adults. As an initial validation for both adults and children, we used retrospectively collected data to demonstrate the models were robust and suitable for use in prospective studies for patients receiving voriconazole. Now, we report the results of our prospective evaluation of the software’s ability to optimally achieve drug exposure targets in patients with hematological malignancy or those undergoing hematopoietic stem cell transplantation.

## RESULTS

**FIG 1:**
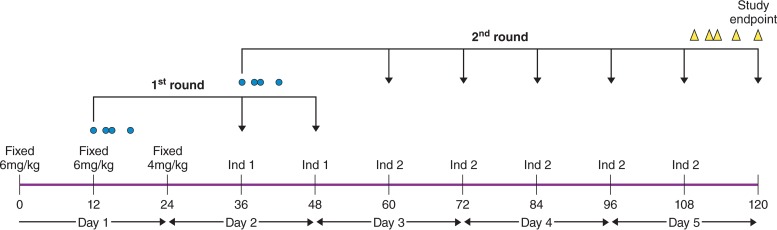
Schema of the study design. The blue dots refer to sampling times for the PK for the first and second rounds of dosage individualization. The yellow triangles represent the sampling strategy to define the primary endpoint. Ind 1 and Ind 2 are the dosage predictions from BestDose in the first and second rounds of dosage individualization, respectively. (Illustration courtesy of Patrick Lane.)

**FIG 2:**
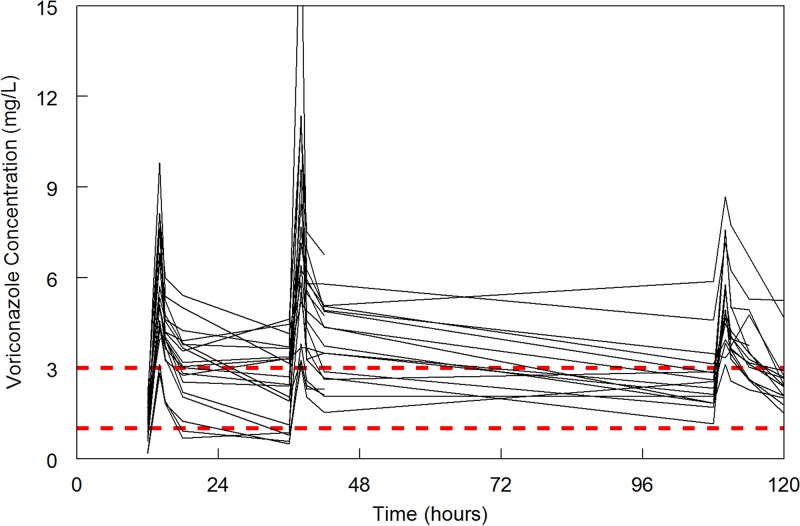
Spaghetti plot showing the PK data for the study patients. The broken red lines indicate the target concentration range of 1 to 3 mg/liter. A total of 12/14 patients achieved a *C*_min_ within the range 1 to 3 mg at 120 h.

The demographics of the study population are summarized in Table 1. Notably, there were no patients from South East Asian countries where there is a high prevalence of pharmacogenetic determinants of slow clearance. A total of 19 patients were recruited to the study (Fig. 1). Of these, 4 were not evaluable for the primary endpoint (1 withdrew consent, and for 3 there were hardware failures of liquid chromatography-tandem mass spectrometry [LC-MS/MS] that meant voriconazole could not be measured). For a further 1 patient, a final sample at 120 h was hemolyzed and unsuitable for analysis. Of the remaining 14 patients, 12 (12/14, 85.7%) had a voriconazole concentration at 120 h within the range 1 to 3 mg/liter. The 2 patients that missed the 1- to 3-mg/liter target both had high concentrations of 4.66 and 5.25 mg/liter. The concentration-time data from the trial for the 14 evaluable patients are shown in Fig. 2.

**TABLE 1:** Demographics

Demographic	Result
Age (IQR)	57 (52–61)
Sex, *n* (%)	Male, 13 (68)
Ethnicity, *n* (%)	
Caucasian	18/19 (95)
Other	1/19 (5)^*a*^
Median ht, cm (IQR)	170 (164–177.5)
Median wt, kg (IQR)	83.00 (69.70–95.80)
Median creatinine level, μmol/liter (IQR)	80 (69.5–98.5)
Diagnosis at enrollment, *n* (%)	
Proven fungal infection	0/19
Probable fungal infection	0/19
Possible fungal infection	0/19
Patient at risk of invasive fungal infection	19/19 (100)

a Other: Brazilian.

A 95% confidence interval from the exact single proportion test from the sample proportion of 12/14 successes (0.857) was 0.572 to 0.982. Hence, even though the number of evaluable patients did not reach the requisite number for the interim analysis (*n* = 18), the very large effect of dosage individualization resulted in a statistically significant outcome from only 14 evaluable patients.

The primary endpoint was further supported by PK/pharmacodynamic (PD) modeling from patients within the trial. A population PK model was fitted to the data from all patients enrolled in the study (*n* = 19). The model fitted the data well with a coefficient of determination (*r*^2^) of 0.71 for the linear regression of the observed-predicted values after the Bayesian step. The parameters are summarized in Table 2. The population PK model was used to estimate each patient’s PK parameters, and these were then used to estimate the anticipated drug exposure resulting from the use of a standard licensed regimen. The mean ± the standard deviation of the directly observed *C*_min_ concentrations from the 14 patients in the study was 2.69 ± 1.04 mg/liter with a range of 1.5 to 5.25 mg/liter. In contrast, the model-predicted *C*_min_ ± standard deviation at 120 h values for the same patients receiving standard fixed intravenous (i.v.) dosing was 5.318 ± 4.39, with a range of 0.09 to 13.81 mg/liter; when considering all 19 patients with predictions available from the population PK model, the number of patients receiving standard fixed i.v. dosing that would have been predicted to achieve 1 to 3 mg/liter was only 3/19 (15.8%), which was less than the *a priori* prediction of 33%.

**TABLE 2:** Parameter values from final population PK model

Parameter (U)^*a*^	Mean	Median	95th credibility limits	SD	CV%^*b*^
*V*_max_ (mg/h)	35.397	30.069	23.533–41.278	20.845	58.889
*K_m_* (mg/liter)	2.129	1.181	0.140–3.924	2.361	110.874
*V* (liters)	45.407	44.933	25.206–66.183	22.901	50.436
*k*_cp_ (h^−1^)	3.308	0.948	0.461–2.494	7.320	221.251
*k*_pc_ (h^−1^)	1.447	0.262	0.169–0.346	4.109	284.065

a *V*_max_ is the maximum rate of enzyme activity (mg/liter), *K_m_* (mg/liter) is the concentration of voriconazole at which enzyme activity is half maximal; *V* is the volume of the central compartment (in liters); and *k*_cp_ and *k*_pc_ (h^−1^) are the first-order intercompartmental rate constants.

b CV%, percent coefficient of variation.

There were no patient deaths in the study up to day 35 postenrollment. Similarly, there were no episodes that were attributed to voriconazole-related toxicity. Hence, no relationships between dosage individualization and secondary outcomes could be established.

Dosage individualization resulted in lower mean drug requirements per patient. The mean (median) ± the standard deviation voriconazole requirements for 10 dosages was 3,073 (2,937) ± 535 mg. The use of a standard fixed weight-based regimen (i.e., 6 mg/kg every 12 h [q12h] for two dosages, followed by 4 mg/kg q12h for 120 h) without therapeutic drug monitoring would have resulted in 3,414 (3,380) ± 737 mg (*P* = 0.174; *t* test). Because of some uncertainty as to whether the data were normally distributed given the relatively small number of observations, a sensitivity analysis using a nonparameteric (Mann-Whitney) test was also performed, which gave similar conclusions (*P* = 0.22).

**FIG 3:**
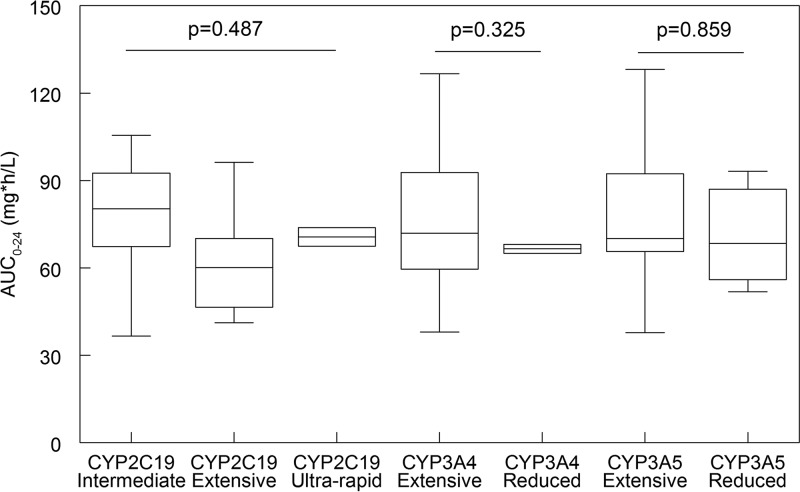
Relationship between AUC_0–24_ in the initial 24 h of voriconazole therapy and genotype. The medians, interquartile ranges (IQR), and 5th and 95th percentiles are shown in each box plot.

The pharmacogenetic genotypes for each patient are shown in Table 3. For one patient, DNA could not be amplified to assess CYP2C19*17 genotype despite multiple attempts. One patient refused consent, meaning that there were 18/19 patients with samples available for pharmacogenetic analyses and 17/19 for CYP2C19*17. There were no statistically significant relationships between the AUC_0–24_ in the initial 24 h of therapy (i.e., all patients received 6 mg/kg q12h for two dosages) and the different genotypes. For *CYP2C19*, the means ± the standard deviation AUC_0–24_ for the intermediate, extensive, and ultrarapid metabolizers were 77.94 ± 20.80, 59.06 ± 18.25, and 70.60 ± 4.55 mg ⋅ h/liter, respectively. The *P* values for the Mann-Whitney U test for the comparisons between the AUC_0–24_ for the phenotypes of *CYP2C19*, *CYP3A4*, and *CYP3A5* were 0.487, 0.325, and 0.859, respectively (Fig. 3). The genotype was not used for dosage individualization because this would require a population PK model with the genotype incorporated as a covariate. Furthermore, there are too few data in this study to enable the construction of new software that could utilize both genotype and voriconazole concentrations.

**TABLE 3:** Details of the *CYP2C1*9, *CYP3A4*, and *CYP3A5* genotypes and voriconazole AUC_0–24_ values in the initial 24 h of therapy

Patient	AUC_0–24_^*a*^	*CYP2C19*^*c*^					*CYP3A4*^*d*^		*CYP3A5*^*e*^	
		CYP2C19*2 (decreased activity)	CYP2C19*3 (decreased activity)	CYP2C19*17 (increased activity)	*2C19* genotype	*2C19* phenotype	CYP3A4*22 (decreased activity)	3A4 group	CYP3A5*3 (decreased activity)	3A5 group
VOR0001	80.24	*1/*2	*1/*1	*1/*1	*1/*2	Intermediate	*1/*1	Extensive	*1/*1	Extensive
VOR0002	70.07	*1/*1	*1/*1	*1/*1	*1/*1	Extensive	*1/*1	Extensive	*1/*1	Extensive
VOR0003	105.43	*1/*2	*1/*1	*1/*17	*2/*17	Intermediate	*1/*1	Extensive	*1/*1	Extensive
VOR0004	67.38	*1/*1	*1/*1	*1/*17	*1/*17	Ultrarapid	*1/*1	Extensive	*1/*1	Extensive
VOR0005	68.43	*1/*1	*1/*1	*1/*1	*1/*1	Extensive	*1/*1	Extensive	*1/*3	Poor
VOR0006	68.12	*1/*2	*1/*1	*1/*17	*2/*17	Intermediate	*1/*22	Poor	*1/*1	Extensive
VOR0007	36.65	*1/*2	*1/*1	*1/*1	*1/*2	Intermediate	*1/*1	Extensive	*1/*1	Extensive
VOR0008^*b*^	135.70	*1/*1	*1/*1	Missing	*1/*1	Unknown	*1/*1	Extensive	*1/*1	Extensive
VOR0009	96.25	*1/*1	*1/*1	*1/*1	*1/*1	Extensive	*1/*1	Extensive	*1/*1	Extensive
VOR0010	93.20	*1/*2	*1/*1	*1/*1	*1/*2	Intermediate	*1/*1	Extensive	*3/*3	Poor
VOR0011	41.21	*1/*1	*1/*1	*1/*1	*1/*1	Extensive	*1/*1	Extensive	*1/*1	Extensive
VOR0012	68.46	*1/*2	*1/*1	*1/*1	*1/*2	Intermediate	*1/*1	Extensive	*1/*1	Extensive
VOR0013	46.50	*1/*1	*1/*1	*1/*1	*1/*1	Extensive	*1/*1	Extensive	*1/*1	Extensive
VOR0014	92.29	*1/*2	*1/*1	*1/*1	*1/*2	Intermediate	*1/*1	Extensive	*1/*1	Extensive
VOR0015	51.76	*1/*1	*1/*1	*1/*1	*1/*1	Extensive	*1/*1	Extensive	*1/*3	Poor
VOR0016	73.82	*1/*1	*1/*1	*1/*17	*1/*17	Ultrarapid	*1/*1	Extensive	*1/*1	Extensive
VOR0017	64.97	*1/*2	*1/*1	*1/*1	*1/*2	Intermediate	*1/*22	Poor	*1/*1	Extensive
VOR0018	92.14	*1/*2	*1/*1	*1/*1	*1/*2	Intermediate	*1/*1	Extensive	*1/*1	Extensive

a The AUC_0–24_ was determined from the population PK model in the inintial 24 h of dosing.

b DNA from this patient could not be amplified to determine the 2C19 status.

c The CYP2C19*2, CYP2C19*3, and CYP2C19*17 genotypes are shown for each patient, and the resulting genotype and phenotype are classified as intermediate, extensive, or ultrarapid.

d The CYP3A4*22 genotype is shown, as well as the resulting genotype and phenotype, classified as extensive and poor.

e The CYP3A5*3 genotype is shown, as well as the resulting genotype and phenotype, classified as extensive and poor.

## DISCUSSION

The multiple model approach that is used by BestDose to achieve dosage individualization is based on two fundamental principles. The first provides a way to summarize past experiences of a drug’s PK. This is achieved using a previously described population PK model (with prospective validation [10]) that is solved on multidimensional grid, which is sized according to the structure of the chosen pharmacokinetic model (10, 11). Nodes or “support points” within this grid represent sets of PK parameters that have the potential to explain the observed PK values obtained from patients within the study population. Sets of parameters that describe data well are more probable, whereas those that do so poorly are downweighted. The collection of active grid points from all patients constitutes a joint probability density for the population. The distribution of probabilities within the grid is unique to the drug and the patient population from which the PK was obtained. For the purposes of dosage individualization, the grid constitutes the Bayesian prior.

The intensive sampling (four samples for each iteration of dosage individualization; eight samples in all) enabled the inherent PK variability of voriconazole to be explicitly demonstrated. With fewer samples, the behavior of voriconazole appears inscrutable with excursions from low to high concentrations and vice versa without apparent reason. This seemingly erratic behavior is a result of the nonlinear PK. As voriconazole concentrations approach and then exceed *K_m_*, clearance is saturated. In this phase, only a fixed amount of drug is metabolized per unit time, and plasma concentrations rise rapidly with the repeated administration of drug. Similarly, if drug is withheld, concentrations may fall precipitously as clearance mechanisms become unsaturated. Our study shows that most of the PK variability is completely predictable and not a result of changes in PK secondary to physiological instability. Consequently, voriconazole is a compound that lends itself to dosage individualization.

The well-described nonlinear PK of voriconazole presents a challenge for dosage individualization. The population estimates for adult patients for the maximum rate of activity for the enzymes involved in voriconazole metabolism (*V*_max_) and the voriconazole concentration at which that enzyme activity is half-maximal (*K_m_*) are 35.39 mg/h and 2.129 mg/liter, respectively. However, the *V*_max_-*K_m_* pair for an individual patient may be completely different from population means and median values (this variability is reflected in the high percent coefficient of variation (CV%) for these parameters; see Table 2). Some patients receiving similar amounts of drug have linear PK, while others have overtly nonlinear PK. Accurate control of patients whose plasma concentrations traverse *K_m_* is difficult without prior knowledge of an individual’s *V*_max_-*K_m_* pair. These values cannot be predicted *a priori* from covariates such as the pharmacogenetic determinants of clearance. Rather, these values can only be estimated by observing the transition from linear to nonlinear PK, which is triggered by dosage escalation. In this study, the majority of patients had high voriconazole concentrations after initial i.v. therapy and exhibited nonlinear PK. These patients subsequently required careful dosage reduction to bring them back into the target concentration range of 1 to 3 mg/liter at the end of 5 days of therapy. Our successful target attainment rate in 85.7% of patients using Bayesian adaptive control was significantly better than a rules-based approach that resulted in a 54% target attainment (8).

This study has several limitations. The study was relatively small and contained patients at the early phases of hematopoietic stem cell transplantation rather than those further into their course where critical illness may have resulted in more variable and extreme pharmacokinetics. The relatively short duration of the study did not expose the potential problems of autoinduction of clearance that has been described (3) or other time-dependent nonlinearities in the PK. Similarly, the study duration was short enough that the PK did not change appreciably as a result of changing clinical state and underlying disease(s) that could have affected clearance and the volume of distribution. This could potentially be circumvented in clinical practice by only providing the immediate past concentrations for dosage calculation. The sampling was relatively intensive and perhaps greater than would be possible in routine clinical practice. However, this was required to demonstrate proof-of-principle. Dosage individualization is perfectly feasible with fewer samples, although there will always be a tradeoff between the intensity of sampling and the precision of dosage optimization. Finally, there was no information related to the oral formulation that is widely used in clinical practice. Given the trial stopped before the intended number of evaluable patients were recruited there is potential for bias if the decision to stop was based upon the emerging result at that time. However, although the trial was unblinded, the decision to stop was driven purely by operational issues.

The effect of dosage individualization was so large that a statistically significant result was obtained with relatively few patients. The study shows that it is possible to achieve precise control for a compound with significant pharmacokinetic variability and nonlinear PK. The question of when a steady state is achieved, which is critical to traditional trough (or *C*_min_)-based therapeutic drug monitoring strategies and dose adjustment becomes irrelevant when using the approach used in this study. Dosage individualization has the potential to markedly reduce the numbers of patients at risk of concentrations that increase the risk of concentration-dependent therapeutic failure, as well as drug toxicity.

## MATERIALS AND METHODS

This was a single-center open-phase II clinical trial in adults ≥18 years with EudraCT number 2013-0025878-34 and ISRCTN number ISRCTN83902726. The study was approved by the Regional Ethics Committee (14/NW/1323). Informed consent was obtained from all patients prior to enrollment. The study was conducted in the Royal Liverpool Broadgreen University Hospitals Trust, which is a large inner city tertiary hospital.

The target concentration for dosage individualization was a *C*_min_ of 2 mg/liter, and this was chosen as a safe and effective target based on a multitude of clinical and nonclinical data (7). The primary endpoint was the proportion of patients that achieved a voriconazole *C*_min_ of 1 to 3 mg/liter at the end of day 5 of therapy (i.e., at 120 h after therapy initiation or 10 dosages of voriconazole). This range was chosen to test the performance of the software and is distinct from the usual concentration range that is used for therapeutic drug monitoring in patients. Secondary endpoints were patients with drug-related toxicity and mortality at 35 days after therapy initiation. Safety was assessed at days 14 and 35.

The null hypothesis was that the proportion of patients reaching the target *C*_min_ range of 1 to 3 mg/liter (*p*) was *P* < 0.33, which was based on simulations from previously fitted population PK models to voriconazole PK data from healthy volunteers and patients (3). The alternative hypothesis was given by an increase in *p* to *P* > 0.67. Using a two-stage design, an initial 18 patients were to be recruited. If ≥6/18 (33%) patients had a trough concentration within the target range (*C*_min_ at 120 h of 1 to 3 mg/liter), the trial was planned to continue onto a second phase. After the second phase, the null hypothesis (*P* < 0.33) would be rejected if >16/33 (48%) patients obtained the target trough concentration. However, due to operational feasibility issues it was necessary to close this study after 19 patients had been recruited, of which 14 patients were evaluable. At the this point, the data were analyzed using the prespecified approach for the final analysis.

### Clinical study.

All patients received i.v. voriconazole (*V_f_*_-end_) at the direction and discretion of the treating clinician for patients either with an invasive fungal infection (with various degrees of certainty), empirically or as prophylaxis at the beginning of hematopoietic stem cell transplantation. An initial dosage of 6 mg/kg every 12 h (q12h) i.v. was administered for two dosages and infused i.v. over 2 h. The loading regimen was followed by a single dosage of 4 mg/kg.

Inclusion criteria were as follows: (i) adults ≥18 years old, (ii) patients initiating voriconazole for suspected or confirmed invasive aspergillosis or other serious fungal infections deemed by the treating physician to be susceptible to voriconazole, (iii) available venous access to permit the administration of voriconazole and procurement of multiple plasma samples to measure voriconazole concentrations; (iv) estimated glomerular filtration rate of ≥50 ml/min using the modified diet renal disease equation, (v) able to give written informed consent, (vi) considered fit to receive the trial treatment, (vii) able to remain in the hospital for at least 5 days, and (viii) not pregnant and/or using appropriate contraception to prevent pregnancy.

The exclusion criteria were as follows: (i) patients receiving any form of renal replacement therapy (i.e., hemodialysis or hemofiltration); (ii) liver enzymes if >3× the upper limit of normal with no evidence of hepatic insufficiency; (iii) female patients who were pregnant, breast feeding, or planning pregnancy during the study; (iv) history of intolerance to voriconazole; (v) evidence of a clinically relevant fungal isolate that was resistant to voriconazole; (vi) QT prolongation of >450 ms; (vii) patients receiving any other medications that were contraindicated with the use of voriconazole (e.g., terbinafine, long acting barbiturates, and ergot alkaloids); (viii) uncontrolled cardiac, respiratory, or other disease or any serious medical or psychiatric disorder that would preclude trial therapy or informed consent; and (ix) hypersensitivity to voriconazole, its excipients or other triazoles.

### Software.

The software used in this study is called BestDose (www.lapk.org) and has previously described (10). For the purposes of this study, BestDose was designated a device by the Medicines and Healthcare products Regulatory Agency (MHRA), which is the United Kingdom’s competent body that regulates medicines and medical devices. The software was compliant with ISO 14971 (risk management of medical device software) and IEC 62304 (guidance on aspects of medical device software required for safe use for patients). The voriconazole model was implemented on a central PC with remote-access dial-in to allow dosage individualization to occur anywhere that an Internet connection was available. All access was password protected and all runs were captured, time and date stamped, and stored securely.

### Sampling and dose adjustment.

The study design is outlined in Fig. 1. The duration of the study to determine the primary endpoint was 120 h, even though patients were followed for 28 days to determine secondary endpoints of mortality and toxicity. The schedule of voriconazole administration was fixed at 12 h. Drug was infused i.v. over 2 h. A fixed licensed dosage was used for the first three dosage intervals (i.e., 6 mg/kg twice as a loading regimen, followed by 4 mg/kg i.v.). Sampling began at the end of the first dosing interval prior to the administration of the second dose (*t* = 12 h) and then at the end of the infusion of the second dose (*t* = 14 h), as well as at 15 h and 18 h. These measurements were then sent as a batch to measure voriconazole concentrations using LC-MS/MS. The dosing information and voriconazole concentrations were then used to calculate the dosing for the fourth and fifth individualized dosages, which were administered at 36 and 48 h.

A second round of dosage individualization was then performed. Blood samples were collected at 36 h (predose), 38 h (end of infusion), 39 h, and 42 h. This batch of four samples were processed in the same manner described above. The PK data were entered into the software and used to plan the sixth, seventh, eighth, ninth, and tenth dosages, which were administered at 60, 72, 84, 96, and 108 h, respectively (Fig. 1). A final set of blood samples were collected at 108 h (predose) and then at 109, 110, 114, and 120 h.

To design an individualized regimen, the patient’s plasma PK values were entered. A target *C*_min_ of 2 mg/liter was used for each dosage interval. If the predicted dosage was >6 mg/kg, a value of 6 mg/kg was fixed, and subsequent dosages were calculated. If the predicted dosage was 0 mg, a minimum fixed dosage of 50 mg was administered, and subsequent dosages were calculated.

### Measurement of voriconazole.

Voriconazole plasma concentrations were measured in real-time using an Agilent 6420 triple quad mass spectrometer. A total of 20 μl of sample was mixed with 300 μl of phenacetin dissolved in acetonitrile, which was used as the internal standard. The mixture was vortexed and centrifuged before injecting 1 μl of sample for analysis. An Agilent Zorbax Eclipse Plus C18+UHPLC Guard (2.1 mm by 50 mm by 1.8 μm) was used, with starting concentrations of 95 and 0.1% aqueous formic acid and 5 and 0.1% formic acid in acetonitrile and a gradient elution of 70:30. The mass transitions for voriconazole were 350 to 281.1 and 350 to 224, whereas the mass transitions for phenacetin were 180.1 to 110 and 180.1 to 65.1. The inter- and intrarun variation was <8%. The stability of voriconazole in whole blood and plasma for 1 h was established. The assay was linear over the dynamic range 0.025 to 20 mg/liter. The limit of quantification was 0.025 mg/liter.

### Population model of voriconazole.

A population PK model was fitted *post hoc* to the combined voriconazole data set from 19 patients enrolled in the study to all the dosing and concentration data at the end of the study. The same structural model as previously described (3) was used. A nonlinear term for clearance was used. A nonparametric population methodology was used, and all fitting was performed using the program Pmetrics (12). The data were weighted by the inverse of the estimated assay variance. The fit of the model to the data was assessed using a visual inspection and linear regression of observed-predicted values both before and after the Bayesian step. Measures of bias and imprecision were assessed.

A population model was used to estimate drug exposure in individual patients receiving a standard licensed weight-based dosage of voriconazole (i.e., 6 mg/kg q12h for two dosages, followed by 4 mg/kg q12h thereafter). Drug exposure was quantified in terms of the *C*_min_ at 120 h posttreatment for the primary endpoint. AUC_0–24_ in the first 24 h was used to assess any potential impact of genotype.

### Genotyping for cytochrome P450 enzyme gene polymorphisms.

Genomic DNA was extracted from 1 ml of whole blood using an E.Z.N.A DNA blood minikit (Omega Bio-Tek, Norcross, GA) according to the manufacturer’s instructions. Twenty nanograms of DNA per reaction was genotyped for CYP2C19*2, CYP2C19*3, CYP2C19*17, CYP3A4*22, and CYP3A5*3 genetic variants using commercially available TaqMan real-time PCR drug metabolism single nucleotide polymorphism assays with 1× TaqMan genotyping mastermix using an ABI 7900HT real-time PCR system according to the manufacturer’s protocol (Applied Biosystems, Carlsbad, CA). All samples were genotyped in duplicate to ensure sample concordance.

For CYP2C19, patients were classified as intermediate (*1/*2, *1/*3, and *2/*17), extensive (*1/*1), or ultrarapid metabolizers (*1/*17 or *17/*17). For CYP3A4, patients were classified as extensive (*1/*1) or poor (*1/*22). Similarly, for CYP3A5, patients were classified as extensive (*1/*1) or poor (*1/*3) metabolizers.
